# GOLD-Induced Cytokine (GOLDIC): A Game-Changer Orthobiologic in Regenerative Medicine

**DOI:** 10.7759/cureus.45435

**Published:** 2023-09-18

**Authors:** Madhan Jeyaraman, Naveen Jeyaraman, Pothuri Rishi Ram, Ravichandran Venkatasalam, Sankalp Yadav

**Affiliations:** 1 Orthopedics, ACS Medical College and Hospital, Dr. MGR Educational and Research Institute, Chennai, IND; 2 Orthopedics and Trauma, Sanjay Gandhi institute of Trauma and Orthopedics, Bengaluru, IND; 3 Regenerative Medicine, Orange Healthcare, Chennai, IND; 4 Medicine, Shri Madan Lal Khurana Chest Clinic, New Delhi, IND

**Keywords:** goldic, regenerative medicine treatments, gelsolin, cytokine, gold

## Abstract

Due to the Industry 4.0 and Industry 5.0 revolutions, researchers, clinicians, and regenerative medicine experts are exploring the plausibility of regenerating diseased or degenerated tissues to regain their near-normal biomechanical properties. In the past three decades, research on “Tissue Engineering and Regenerative Medicine” (TERM) has attained various milestones in clinical translation from bench to bedside. The regulatory bodies of various countries and states are working on the ethical use and guidelines for the production and storage of various cellular and acellular products. Platelets and platelet-derived by-products play a significant role in TERM. The growth factors and cytokines present in platelets regenerate the tissue of interest. In this connotation, a newer orthobiologic called “GOLD-induced cytokine” (GOLDIC) has become a product of interest among various regenerative medicine experts and researchers around the globe. Due to its potent anti-inflammatory action and potential systemic side effects, gold has been withdrawn from the management panel for rheumatoid arthritis. With the knowledge of its anti-inflammatory properties, researchers explored the utility of gold for tissue regeneration.

## Editorial

In the dynamic confluence of cutting-edge technological advancements and regenerative medicine, the convergence of Industry 4.0 and Industry 5.0 has catalyzed a transformative breakthrough known as GOLD-induced cytokine (GOLDIC) therapy. This innovative paradigm presents a renewed beacon of hope for patients grappling with degenerative diseases and tissue injuries by harnessing the intricate interplay of platelets and their derived products. Through orchestrating tissue regeneration and adept management of inflammation-driven pathologies, GOLDIC therapy stands as a vanguard in medical innovation.

Historical genesis

The roots of GOLDIC therapy can be traced back to the visionary German physicist Ulrich Schneider, who unveiled the latent potential of hydrophilic gold particles within tissue engineering. This revelation marked the inception of a symphony of intricate molecular responses that yield a serum teeming with anti-catabolic and anabolic factors, constituting the fundamental tissue regeneration bedrock. The revival of gold, once associated with rheumatoid arthritis management but relegated due to systemic side effects, symbolizes the resilience of scientific exploration and the transformative potency of novel perspectives.

Evolutionary trajectory

GOLDIC therapy emulates the harmonious convergence of material science, biomedical engineering, and clinical innovation. Its evolutionary trajectory underscores a remarkable odyssey from laboratory discovery to tangible patient-centric benefits. Through the amalgamation of Industry 4.0 and Industry 5.0 technologies with the principles of regenerative medicine, novel therapeutic paradigms have transcended the boundaries of erstwhile scientific conjecture, propelling the frontiers of tissue engineering and regenerative medicine.

Mechanistic ingenuity

At the core of GOLDIC therapy lies the exquisite orchestration of platelets and their ensuing products. Hydrophilic gold particles are instigators, creating a harmonious ensemble of molecular cascades that culminate in a serum enriched with a panoply of growth factors and cytokines. This potent cohort synergistically expedites tissue regeneration while displaying formidable anti-inflammatory attributes. At the heart of this therapeutic approach lies an intricate mechanistic interplay, elucidating how platelets and hydrophilic gold particles orchestrate a transformative response for tissue regeneration and inflammation management [1]. This serum emerges as a paradigm-shifting therapeutic elixir, divided from gold compounds.

Steps in preparation for GOLDIC

Synthesis of hydrophilic gold particles; blood culturing and interaction with hydrophilic gold particles; platelet activation and growth factor release; serum collection and processing

GOLDIC treatment starts with taking a sample of the patient’s blood. This blood is now prepared using GOLDIC tubes, which include specially designed gold particles and a special filter to prevent these particles, cells, and platelets from being injected with the processed, enriched in cytokines and growth factors serum. During the incubation process (24 hours), gold particles act as catalysts for monocytes to produce the whole spectrum of autologous cytokines, which is the most important part of the process. The GOLDIC tubes filled with patients’ blood after the incubation will be centrifuged to separate serum from the other blood components. This serum, enriched with growth factors and important cytokines, is then provided for injection.

Quality control and standardization

Rigorous quality control measures are employed to ensure the consistency and potency of the serum obtained from different patient samples. This step involves assessing the concentrations of key growth factors and cytokines to guarantee the therapeutic efficacy of the final product.

Clinical administration

The prepared serum, which is now abundant in bioactive molecules capable of promoting tissue regeneration and modulating inflammation, is administered to the patient. Depending on the medical condition being treated, the serum may be delivered directly to the target tissue or systemically through an intravenous infusion.

Once introduced into the bloodstream, the hydrophilic gold particles serve as initiators of a multifaceted cascade of molecular events. Their unique surface characteristics facilitate interactions with blood components, particularly platelets. This interaction triggers platelet activation, culminating in a complex sequence of intracellular signaling pathways. Within activated platelets, these signaling pathways converge on releasing various bioactive molecules, including growth factors and cytokines. Among the prominent players are platelet-derived growth factor (PDGF), transforming growth factor beta (TGF-β), vascular endothelial growth factor (VEGF), and interleukins (ILs), each contributing to distinct facets of tissue regeneration and immunomodulation. PDGF, for instance, stimulates cell proliferation and migration, promoting tissue repair and regeneration. TGF-β regulates cell differentiation and extracellular matrix deposition, which is crucial for tissue remodeling and wound healing. VEGF augments angiogenesis, fostering the formation of new blood vessels to nourish regenerating tissues. ILs orchestrate immune responses, aiding in the recruitment of immune cells for tissue repair and curtailing inflammation.

The resulting serum, enriched with these potent bioactive molecules, exerts multifunctional effects on the targeted tissues. It accelerates the recruitment and proliferation of progenitor cells necessary for tissue repair, facilitates the remodeling of the extracellular matrix, and enhances vascularization for improved nutrient and oxygen supply. Simultaneously, the serum exerts immunomodulatory effects, dampening excessive inflammation while preserving the measured immune response necessary for healing.

Moreover, the presence of hydrophilic gold particles during these orchestrated events potentiates the therapeutic impact. Their precise role involves modulating signaling pathways and cellular responses, possibly through their unique physicochemical properties and interactions with cellular membranes. However, this promising therapeutic paradigm also presents challenges, particularly with regard to potential side effects (like pain, swelling, recurrence of symptoms, and malignant transformation of benign pathologies) and the need for comprehensive safety assessments. Addressing these concerns through rigorous research and methodologically sound clinical trials is essential to substantiate the efficacy and safety of GOLDIC therapy.

Potential clinical applications

Orthopedic Conditions

The serum rich in growth factors and cytokines could enhance the regeneration of tissue and improve the overall healing process in conditions like plantar fasciosis [2].

Osteoarthritis and Joint Degeneration

The therapy's ability to promote tissue regeneration and control inflammation might help restore joint health and function in conditions like osteoarthritis [3] and degenerative lumbar spinal stenosis [4].

Wound Healing and Dermatology

GOLDIC therapy may be used to expedite wound healing, particularly in chronic wounds and diabetic ulcers. The bioactive molecules in the serum could promote skin cell proliferation, angiogenesis, and extracellular matrix synthesis, leading to faster wound closure.

Cardiovascular Regeneration

The therapy might contribute to cardiac tissue regeneration after heart attacks, potentially improving heart function. The growth factors in the serum could stimulate the growth of new blood vessels and promote the survival of cardiac cells.

Neurological Disorders

GOLDIC therapy could have applications in treating neurodegenerative diseases like Parkinson's or Alzheimer's. The therapy's ability to stimulate cell growth and modulate inflammation might aid in preserving neuronal health and promoting neural regeneration.

Autoimmune Diseases

GOLDIC therapy's anti-inflammatory effects could be harnessed to manage autoimmune diseases like rheumatoid arthritis. The therapy might help alleviate symptoms and improve patient outcomes by modulating immune responses.

Cosmetic and Aesthetic Medicine

The regenerative properties of GOLDIC therapy might find applications in cosmetic and aesthetic medicine. It could enhance skin rejuvenation and tissue remodeling, addressing concerns like wrinkles and sagging skin.

Dental and Maxillofacial Applications

GOLDIC therapy could aid in promoting bone regeneration in dental and maxillofacial procedures, such as dental implant placement and jaw reconstruction.

Tissue Engineering and Regenerative Medicine

Beyond specific clinical conditions, GOLDIC therapy could play a pivotal role in tissue engineering approaches, enhancing the creation of functional tissues for transplantation and other regenerative medicine applications.

Chronic Inflammatory Conditions

The anti-inflammatory properties of GOLDIC therapy might be utilized to manage chronic inflammatory conditions such as Crohn's disease, ulcerative colitis, or psoriasis.

Advantages of GOLDIC

GOLDIC therapy ushers in a dual-edged advantage, effectively fostering tissue regeneration while maintaining a vigilant check on inflammation. Its distinctive ability to harness the inherent recuperative mechanisms of the body while concurrently ameliorating inflammation-driven pathologies sets it apart. This duality begets a versatile spectrum of potential applications, adroitly addressing a diverse panorama of conditions and patient exigencies. Furthermore, the judicious repurposing of gold underscores the therapy's innovative underpinnings, a testament to the perpetual metamorphosis characterizing medical research.

Challenges and considerations

While the prospects of GOLDIC therapy are promising, challenges loom large. The historical affiliation of this therapy with gold in rheumatoid arthritis management mandates meticulous scrutiny of potential side effects and untoward reactions. Prioritizing patient safety, comprehensive comprehension of long-term implications, and diligent addressing of concerns pertaining to gold compounds stand as imperatives. Comprehensive insights via exhaustive research and rigorous clinical trials are non-negotiable to substantiate the safety and efficacy of GOLDIC therapy, as is requisite for avant-garde therapeutic modalities.

Clinical and ethical deliberations

The introduction of pioneering therapies like GOLDIC invariably engenders ethical and regulatory contemplations. Striking a judicious equilibrium between scientific exploration and conscientious innovation assumes paramount significance. Ethical employment, adept production, and secure storage of cellular and acellular derivatives stemming from GOLDIC therapy necessitate vigilant stewardship by regulatory bodies, researchers, clinicians, and policymakers. Anchoring patient well-being, transparency, and alignment with ethical frameworks emerge as cardinal tenets as this therapeutic endeavor advances toward clinical integration.

Prospective trajectory

The horizon for GOLDIC therapy is fraught with boundless promise. Through the collaborative endeavors of researchers, clinicians, and policymakers, the impact of this therapy could transcend tissue regeneration and inflammation modulation. Sustained research efforts are poised to unveil hitherto undiscovered applications, refine mechanistic insights, and expand the therapeutic purview. By deftly navigating ethical complexities and conducting methodologically robust investigations, GOLDIC therapy stands poised to usher in a metamorphic shift in the landscape of regenerative medicine, engendering novel avenues for healing and augmenting patient outcomes.

In the ever-evolving landscape of medical innovation, GOLDIC therapy stands as a remarkable convergence of cutting-edge technology and regenerative medicine. Its journey, from the visionary insights of Ulrich Schneider to its current status on the cusp of clinical application, underscores the profound strides made in the realm of scientific progress. GOLDIC therapy's ability to orchestrate intricate molecular responses through platelets and hydrophilic gold particles, facilitating tissue regeneration and inflammation control, presents a transformative approach to patient care. However, as with any pioneering medical intervention, careful consideration of safety, ethical implications, and rigorous research is paramount. The historical context of gold's role in medical treatments necessitates a meticulous examination of potential adverse effects, reinforcing the ethical commitment to patient welfare and responsible advancement. Looking forward, the potential applications of GOLDIC therapy extend beyond the boundaries of its current scope. Through interdisciplinary collaboration guided by rigorous regulatory oversight and ethical governance, this therapy could redefine the landscape of regenerative medicine. The GOLDIC narrative exemplifies the capacity of science and technology to redefine therapeutic avenues, culminating in a future where healing and regeneration are tangible prospects. As we move ahead, the saga of GOLDIC therapy serves as a testament to the potential of scientific prowess to shape a healthier and more promising world.
